# Development of stroke identification algorithm for claims data using the multicenter stroke registry database

**DOI:** 10.1371/journal.pone.0228997

**Published:** 2020-02-14

**Authors:** Jun Yup Kim, Keon-Joo Lee, Jihoon Kang, Beom Joon Kim, Moon-Ku Han, Seong-Eun Kim, Heeyoung Lee, Jong-Moo Park, Kyusik Kang, Soo Joo Lee, Jae Guk Kim, Jae-Kwan Cha, Dae-Hyun Kim, Tai Hwan Park, Moo-Seok Park, Sang-Soon Park, Kyung Bok Lee, Hong-Kyun Park, Yong-Jin Cho, Keun-Sik Hong, Kang-Ho Choi, Joon-Tae Kim, Dong-Eog Kim, Wi-Sun Ryu, Jay Chol Choi, Mi-Sun Oh, Kyung-Ho Yu, Byung-Chul Lee, Kwang-Yeol Park, Ji Sung Lee, Sujung Jang, Jae Eun Chae, Juneyoung Lee, Hee-Joon Bae, on behalf of the CRCS-K investigators

**Affiliations:** 1 Department of Neurology, Seoul National University Bundang Hospital, Seoul National University College of Medicine, Seongnam, Republic of Korea; 2 Department of Clinical Preventive Medicine, Seoul National University Bundang Hospital, Seoul National University College of Medicine, Seongnam, Republic of Korea; 3 Department of Neurology, Nowon Eulji Medical Center, Eulji University, Seoul, Republic of Korea; 4 Department of Neurology, Eulji University Hospital, Eulji University College of Medicine, Daejeon, Republic of Korea; 5 Department of Neurology, Dong-A University Hospital, Dong-A University College of Medicine, Busan, Republic of Korea; 6 Department of Neurology, Seoul Medical Center, Seoul, Republic of Korea; 7 Department of Neurology, Soonchunhyang University Hospital, Soonchunhyang University College of Medicine, Seoul, Republic of Korea; 8 Department of Neurology, Inje University Ilsan Paik Hospital, Goyang, Republic of Korea; 9 Department of Neurology, Chonnam National University Hospital, Chonnam National University College of Medicine, Gwangju, Republic of Korea; 10 Department of Neurology, Dongguk University Ilsan Hospital, Goyang, Republic of Korea; 11 Department of Neurology, Jeju National University Hospital, Jeju National University School of Medicine, Jeju, Republic of Korea; 12 Department of Neurology, Hallym University Sacred Heart Hospital, Hallym University College of Medicine, Anyang, Republic of Korea; 13 Department of Neurology, Chung-Ang University Hospital, Chung-Ang University College of Medicine, Seoul, Republic of Korea; 14 Clinical Research Center, Asan Institute for Life Sciences, Asan Medical Center, Seoul, Republic of Korea; 15 Department of Biostatistics, Korea University College of Medicine, Seoul, Republic of Korea; University of Ioannina School of Medicine, GREECE

## Abstract

**Background:**

Identifying acute ischemic stroke (AIS) among potential stroke cases is crucial for stroke research based on claims data. However, the accuracy of using the diagnostic codes of the International Classification of Diseases 10^th^ revision was less than expected.

**Methods:**

From the National Health Insurance Service (NHIS) claims data, stroke cases admitted to the hospitals participating in the multicenter stroke registry (Clinical Research Collaboration for Stroke in Korea, CRCS-K) during the study period with principal or additional diagnosis codes of I60-I64 on the 10^th^ revision of International Classification of Diseases were extracted. The datasets were randomly divided into development and validation sets with a ratio of 7:3. A stroke identification algorithm using the claims data was developed and validated through the linkage between the extracted datasets and the registry database.

**Results:**

Altogether, 40,443 potential cases were extracted from the NHIS claims data, of which 31.7% were certified as AIS through linkage with the CRCS-K database. We selected 17 key identifiers from the claims data and developed 37 conditions through combinations of those key identifiers. The key identifiers comprised brain CT, MRI, use of tissue plasminogen activator, endovascular treatment, carotid endarterectomy or stenting, antithrombotics, anticoagulants, etc. The sensitivity, specificity, and diagnostic accuracy of the algorithm were 81.2%, 82.9%, and 82.4% in the development set, and 80.2%, 82.0%, and 81.4% in the validation set, respectively.

**Conclusions:**

Our stroke identification algorithm may be useful to grasp stroke burden in Korea. However, further efforts to refine the algorithm are necessary.

## Introduction

Claims data, as one type of “big data,” have been actively used for medical research by virtue of a large number of cases, a long period of observation, comprehensiveness, and representativeness of data, especially where a national insurance system encompasses the entire population.[1–4] In stroke research, claims data are increasingly being used for epidemiological, quality-of-care, and cost studies.[5–13]

However, there are crucial limitations in previous claims data-based studies with respect to identification of stroke cases.[6–8, 14–20] Most cases missed validation.[6–8, 14–16, 18] This problem is mostly caused by difficulty to validate the process of identifying stroke cases using claims data through the classic epidemiologic methods, such as review of medical records or direct interview of patients or their families due to a large amount of case numbers and accessibility to the patients or their medical records. If these obstacles could be overcome by linkage with the already validated registry database, the use of claims data in stroke research will be escalated.

The Clinical Research Collaboration for Stroke in Korea (CRCS-K) registry is an ongoing multicenter stroke registry covering most areas of Korea.[21] More than 90% of the registered stroke cases have been confirmed with MRI[21], and characteristics of the registered cases were reported to represent the entire nation.[22] The National Health Insurance System (NHIS) is a compulsory government insurance service system subscribed to by all citizens living in South Korea with universal coverage.[23] According to the support of the NHIS, we had an opportunity to evaluate the claims data of more than 40,000 cases discharged with principal or additional diagnosis codes of stroke in the hospitals participating in the CRCS-K registry through the linkage with the registry database.

We intended to develop and validate the stroke identification algorithm for acute ischemic stroke (AIS) using claims codes relating to the diagnosis and treatment of AIS patients during hospitalization. The stroke cases were certified through the linkage between the claims data and the CRCS-K registry database.

## Materials and methods

### Data sources (Fig 1)

**Fig 1:**
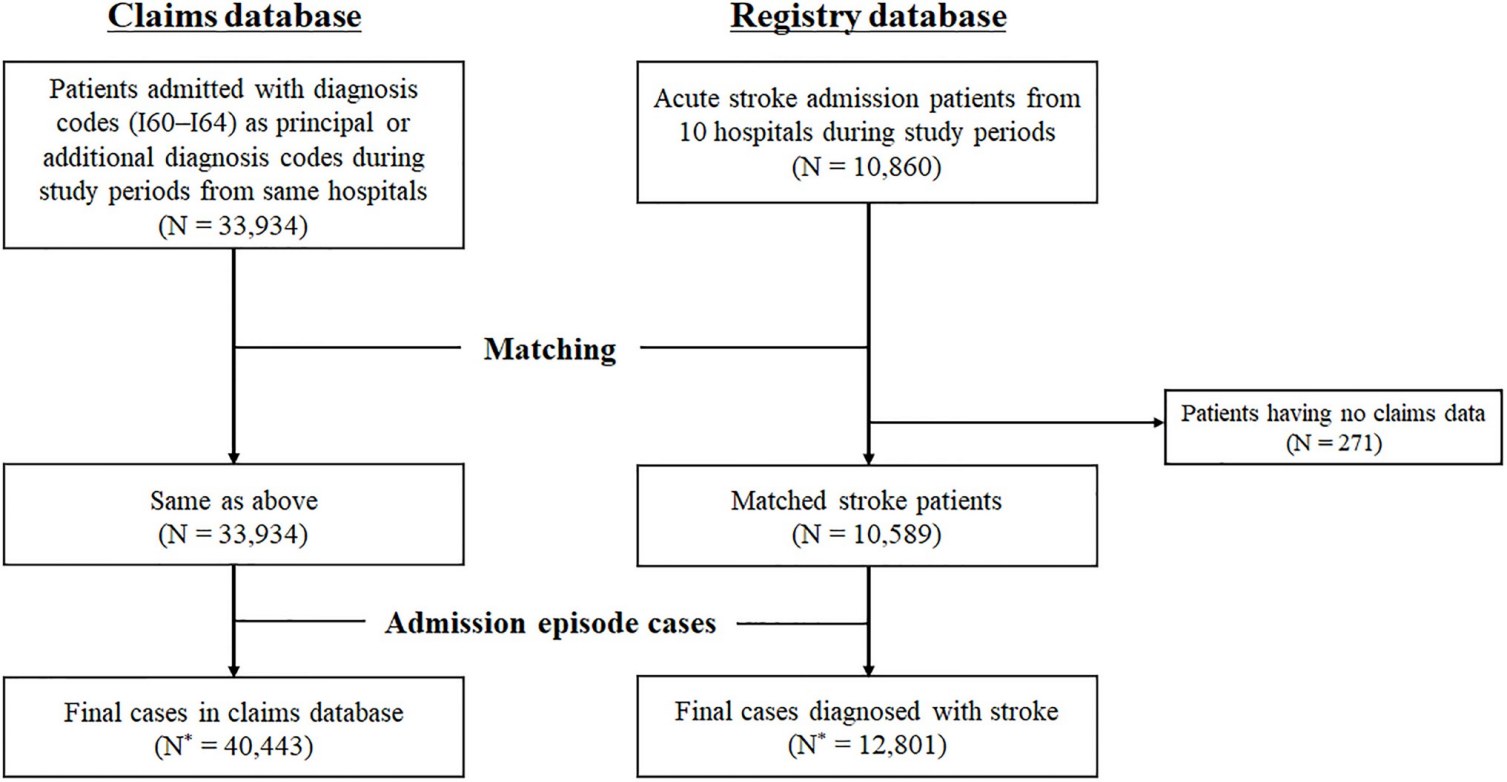
Data sources. ‘N’ and ‘N*’ indicates the number of patients and cases, respectively.

From the NHIS claims data, patients who were admitted to the 10 participating hospitals between 2011 and 2013 with principal or additional diagnosis codes of I60–64 (I60, subarachnoid hemorrhage [SAH]; I61, intracranial hemorrhage [ICH]; I62, other nontraumatic intracranial hemorrhage; I63, cerebral infarction; I64, cerebrovascular disease, not otherwise specified) on the 10th revision of International Classification of Diseases (ICD-10) were selected. Patients who had claims with diagnosis codes of I60–64 before an index stroke were excluded.

Patients, who were hospitalized to the participating hospitals during the study period and registered as AIS in the CRCS-K registry database,[21] were considered the gold standard. The chosen cases were image-proven ischemic stroke patients who were hospitalized within 7 days of onset.[24] During the study period, 92.4% of patients who were enrolled in the CRCS-K registry gave their consent to linking their data to other secondary databases, including the NHIS claims database.

The claims database was linked with the CRCS-K database using the claim serial number, which was generated in each claim at an individual hospital for reimbursement. Stroke cases in the claims database were determined as a true AIS or not depending on the existence of the linkage with the CRCS-K registry database. We excluded patients who were enrolled in the registry database but had no claims data. Following this matching process, the claims and registry database were reconstructed from patient-based to admission episode-based, and through this reconstruction of databases, the number of cases in the claims and registry database increased due to readmission cases. The analysis unit in this study was an admission episode.

The claims database was randomly divided into the development and validation datasets with a ratio of 7:3 for the purpose of internal validation. The developed algorithm for AIS, as described below, was applied to this development dataset and was modified to improve the sensitivity and specificity of the algorithm. The revised final algorithm was applied to the development and validation datasets, and its sensitivity, specificity, accuracy, and predictive values were obtained.

### Clinical workflows of acute ischemic stroke patients in South Korea (S1 Fig)

A majority of AIS patients are admitted to the department of neurology via emergency room in South Korea. Nearly half of them are treated in general wards, about one-thirds in stroke units, and the others in intensive care units [25–27]. The clinical workflows of examinations and treatments which AIS patients receive during hospitalization are shown in S1 Fig. After the acute period, 69% of AIS patients are discharged to home and 23% are transferred to in-hospital rehabilitation services or other long-term care facilities if necessary [27].

### Key identifiers

A working group composed of 14 vascular neurologists developed the algorithm through regular meetings. First, the specific claims codes relating to managing AIS patients with consideration of their frequencies in the claims data were selected, and 17 key identifiers were defined (Table 1 and S1 Fig). Key identifiers consisted of diagnosis codes and other claims codes based on the evaluation of risk factors for stroke, brain imaging, reperfusion therapy, intervention, medication, and rehabilitation for AIS.

**Table 1:** Operational definitions of key identifiers.

No.	Key identifiers	Definition	Periods^†^	KCD Codes^‡^	Contents
1	AF	Principal or additional diagnosis codes of atrial fibrillation among in-hospital and out-patient clinic data	0 days ~ + 30 days	I48, I480, I481, I482, I489	Atrial fibrillation
2	Brain CT	Brain CT claims codes among in-hospital and out-patient clinic data	– 7 days ~ + 2 days	HA451, HA441, HA461, HA851	Brain CT with or without contrast material
				HA471	Brain CT angiography
3	Brain MRI	Brain MR claims codes among in-hospital and out-patient clinic data	– 7 days ~ + 2 days	HE101, HE201, HE301, HE401, HE501	Brain MRI
				HE135, HE136, HE235, HE236, HE535, HE536	Brain or neck MRA
				HF101, HF202	Diffusion MRI
				HF202	Perfusion MRI
4	CTA	Brain CT angiography claims codes among in-hospital and out-patient clinic data	– 7 days ~ + 2 days	HA471	Brain CT angiography
5	Image F/U	Two or more brain imaging codes of key identifiers including brain CT or MRI among in-hospital and out-patient clinic data	– 7 days ~ + 7 days	Same as number 2, 3, 4	
6	Holter	24-hour electrocardiogram monitoring claims codes among in-hospital data	0 days ~ + 30 days	E6545	24-hour Holter monitoring
7	IVT	Medication claims codes of rt-PA among in-hospital data	0 days ~ + 2 days	223501BIJ, 223502BIJ	Alteplase
8	EVT	Endovascular thrombectomy or intra-arterial thrombolysis claims codes among in-hospital data	0 days ~ + 2 days	M6630, M6631, M6632, M6633, M6635	Percutaneous thrombus removal–thrombolysis—intracranial vessel, cerebral, others
				M6636, M6637, M6639	Percutaneous thrombus removal–mechanical thrombectomy–intracranial or extracranial vessels, others
9	CEA	Atherectomy claims codes among in-hospital data	0 days ~ + 30 days	O0226, O0227, O2066	Transluminal atherectomy–carotid artery
10	Carotid angioplasty	Carotid artery angioplasty or stenting claims codes among in-hospital data	0 days ~ + 30 days	M6594	Percutaneous transluminal angioplasty–carotid
				M6602	Percutaneous intravascular installation of metallic stent–carotid
11	Intracranial angioplasty	Intracranial artery angioplasty or stenting claims codes among in-hospital data	0 days ~ + 30 days	M6593, M6597, M6599	Percutaneous transluminal angioplasty–cerebral, others
				M6601, M6605	Percutaneous intravascular installation of metallic stent–cerebral, others
12	New antithrombotics ≤3D	New anti-thrombotic agents that were not used in the last 6 months among in-hospital and out-patient clinic data	0 days ~ + 3 days	110701ATB, 110701ATE, 110702ATB, 110801ATB, 110802ATB, 111001ACE, 111001ACH, 111001ATB, 111001ATE, 111002ATE, 111003ACE, 111003ATE, 256800ATB, 517900ACE, 517900ATE, 517900ACH	Aspirin
				136901ATB, 492501ATB, 495201ATB, 498801ATB, 501501ATB, 517900ACE, 517900ATE, 517900ACH	Clopidogrel
				133201ACR, 133201ATB, 133201ATR, 133201ATD, 133202ATB, 133203ATR, 506100ATB	Cilostazol
				244101ACE, 244101ACH, 244102ACH	Triflusal
				239201ATB, 239202ATB, 498900ATB, 565300ATB	Ticlopidine
				249103ATB, 249105ATB, 249101ATB, 249102ATB, 249104ATB, 249106ATB, 249107ATB, 249108ATB, 249109ATB	Warfarin
				511401ATB, 511402ATB, 511403ATB, 511404ATB	Rivaroxaban
				613701ACH, 613702ACH, 613703ACH	Dabigatran
				617001ATB, 617002ATB	Apixaban
				643601ATB, 643602ATB, 643603ATB	Edoxaban
				152130BIJ, 152131BIJ, 152132BIJ, 152133BIJ, 152134BIJ, 152135BIJ, 152101BIJ, 152102BIJ, 152103BIJ, 152104BIJ, 152105BIJ, 152106BIJ	Enoxaparin
				140230BIJ, 140231BIJ, 140232BIJ, 140233BIJ, 140234BIJ, 140201BIJ, 140202BIJ, 140203BIJ	Dalteparin
				198401BIJ, 198402BIJ, 198403BIJ, 198404BIJ, 198405BIJ, 198406BIJ, 198430BIJ, 198431BIJ, 198432BIJ	Nadroparin
				168630BIJ, 168632BIJ, 168631BIJ, 168601BIJ, 168602BIJ	Heparin
13	New antithrombotics ≤7D	New anti-thrombotic agents that were not used in the last 6 months among in-hospital and out-patient clinic data	0 days ~ + 7 days	Same as above	Same as above
14	New antithrombotics ≤90D at NR/NS OPD	New anti-thrombotic agents prescribed at neurology or neurosurgery OPD that were not used in the last 6 months among in-hospital and out-patient clinic data	0 days ~ + 90 days	Same as above	Same as above
15	Anticoagulants ≤7D	Anticoagulants claims codes among in-hospital and out-patient clinic data	0 days ~ + 7 days	Claims codes of warfarin, rivaroxaban, dabigatran, apixaban, edoxaban, enoxaparin, dalteparin, nadroparin, heparin	
16	Rehab	Rehabilitation procedure codes among in-hospital and out-patient clinic data	0 days ~ + 30 days	MX141, MM301, MM302, MM101, MM102, MM103, MM105, MM111, MM112, MM113, MM114	Rehabilitation
17	Transfer to Rehab	Presence of claims codes prescribed from both neurology and rehabilitation departments in same hospitalization periods	0 days ~ + 30 days	Any claims codes from neurology and rehabilitation department	

^†^Periods for searching claims codes were based on the start date of in-hospital claims data.

^‡^The sixth edition of the Korean Standard Classification of Diseases (KCD), which is based on the tenth revision of the ICD, was used to define each key identifier.

Abbreviations: AF, atrial fibrillation; CTA, CT angiography; F/U, follow up; IVT, intravenous thrombolysis; EVT, endovascular treatment; CEA, carotid endarterectomy; 3D, 3 days; 7D, 7 days; 90D, 90 days; NR, Neurology; NS, Neurosurgery; OPD, out-patient department; Rehab, rehabilitation.

The diagnosis code of *atrial fibrillation (AF)* was selected for the detection of cardioembolic stroke. Among diagnostic evaluations, we chose *brain computed tomography (CT)*, *brain CT angiography (CTA)*, *brain magnetic resonance imaging (MRI)*, and *24-hour electrocardiogram monitoring (Holter monitoring)*. *Brain CTA* was chosen as an independent key identifier to find AIS patients who did not undergo brain MRI and to exclude hemorrhagic stroke patients. A key identifier of *Image follow-up (F/U)* was used to detect patients who were presented with transient ischemic attack (TIA) with no acute lesion on the first CT or MRI but had acute ischemic lesions on re-imaging within 7 days.

A key identifier of *new antithrombotics* was defined (1) if the antithrombotic agent was prescribed for the first time at admission, (2) if a new ingredient of antithrombotic agent was added when patients had antithrombotics prior to index stroke, or (3) if the composition of antithrombotics was changed during admission. As time points for determining the prescription of antithrombotics, 3, 7, and 90 days from stroke onset were chosen. In cases of 90 days from onset, the antithrombotics were confined to those prescribed at neurology and neurosurgery outpatient clinics. Intervention or surgery, including *carotid endarterectomy* or *carotid and intracranial angioplasty/stenting*, can be performed not only in an emergent situation during the acute stage of stroke, but also electively. Therefore, they were used as key identifiers only combined with *new antithrombotics*. A key identifier of *rehab* means receiving rehabilitation therapy during admission due to an index stroke. The transfer of patients from the neurology department to the rehabilitation department during admission due to an index stroke was defined as the *Transfer to Rehab* key identifier.

### Stroke identification algorithm

Using the selected key identifiers, the working group developed the algorithm for identifying AIS based on the clinical flow of hospitalized AIS patients. From the beginning, we divided the algorithm into two scenarios: whether the principal diagnosis code was I63 or not. To find hyperacute ischemic stroke cases, the combination of brain CT and reperfusion therapy (intravenous thrombolysis [*IVT]* or endovascular treatment [*EVT]*) was placed in front of the algorithm. As intervention-related key identifiers have a high specificity, we placed the key identifiers in the front position of the algorithm. Later, brain MRI, medication, and rehabilitation-related key identifiers were placed to identify cases that did not undergo reperfusion therapy or intervention.

The algorithm initially developed as above was applied to the development dataset and was modified by changing the position of the key identifiers or adding a new key identifier to it.

#### Statistical analysis

As described above, AIS cases in the CRCS-K database were considered the “gold standard” with which the results of applying the developed algorithm to the claims database was validated. Sensitivity, specificity, accuracy, positive predictive value (PPV), and negative predictive value (NPV) were calculated for the development and validation datasets. Statistical analyses were performed using SAS version 9.4 (SAS Institute Inc, Cary, North Carolina).

### Ethics statements

The collection of clinical information for the CRCS-K registry and the linkage of this collected information with secondary databases for the purpose of stroke research with informed consent were approved by the local ethics committees of all the participating centers. The use of the CRCS-K database and its linkage with the NHIS claims database for this study was approved further by the Institutional Review Board of Seoul National University Bundang Hospital (No. B-1511/322-106).

## Results

**Fig 2:**
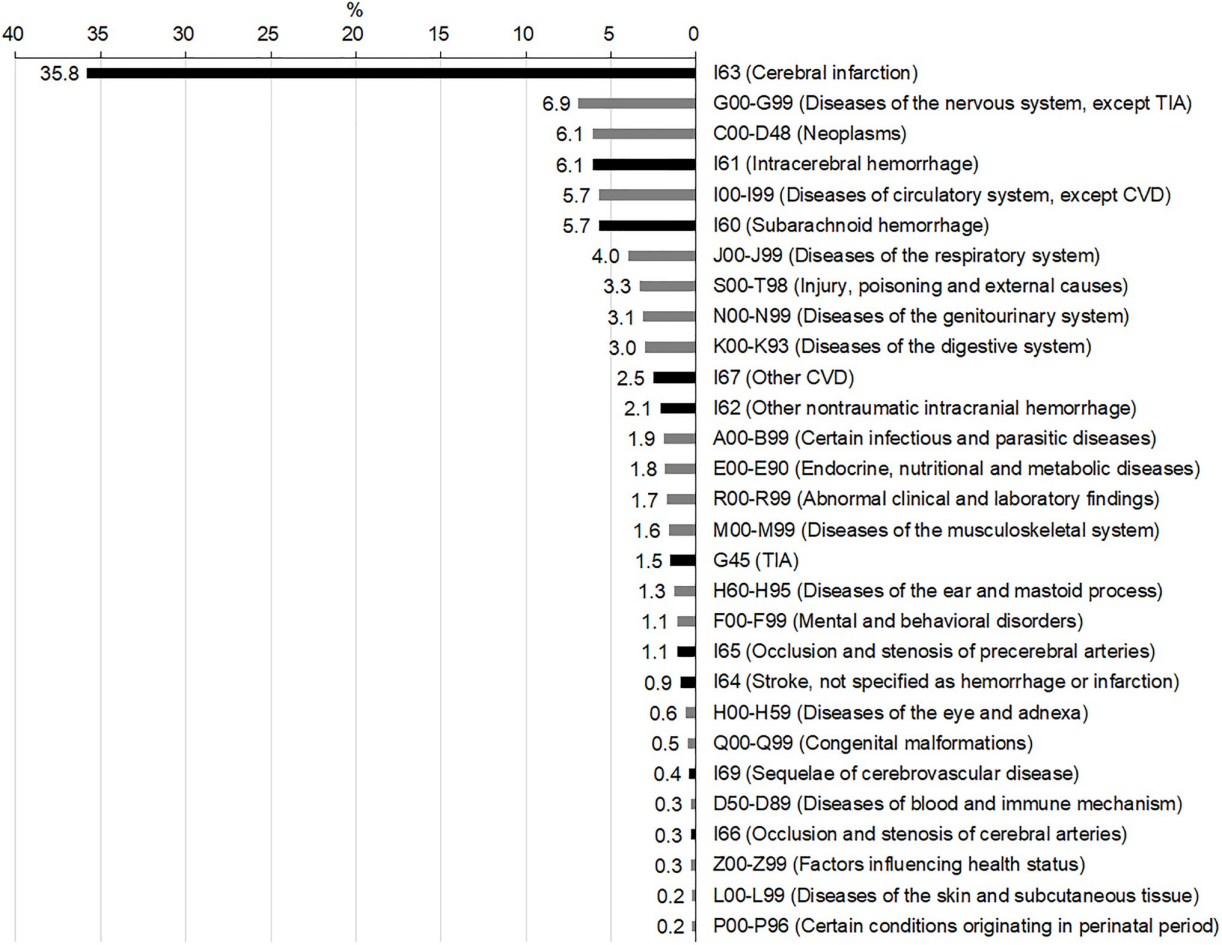
Proportions of principal diagnosis codes at admission. Abbreviations: TIA, transient ischemic attack; CVD, cerebrovascular diseases.

Altogether, 40,443 admission episodes with ICD-10 I60–I64 as principal or additional diagnosis codes were analyzed (Fig 1). Fifty-four percent of this population was male (n = 21,920), and the mean age was 65.6 ± 15.6 years. Among these, 12,801 (32%) episodes were successfully linked to the CRCS-K registry database and identified as true AIS. When analyzing the proportions of principal diagnosis codes for these 40,443 episodes (Fig 2), one-third were cerebral infarction, followed by other nervous system diseases (6.9%), neoplasms (6.1%), ICH (6.1%), other circulatory system diseases (5.7%), and SAH (5.7%). TIA accounted for approximately 1.5%.

**Fig 3:**
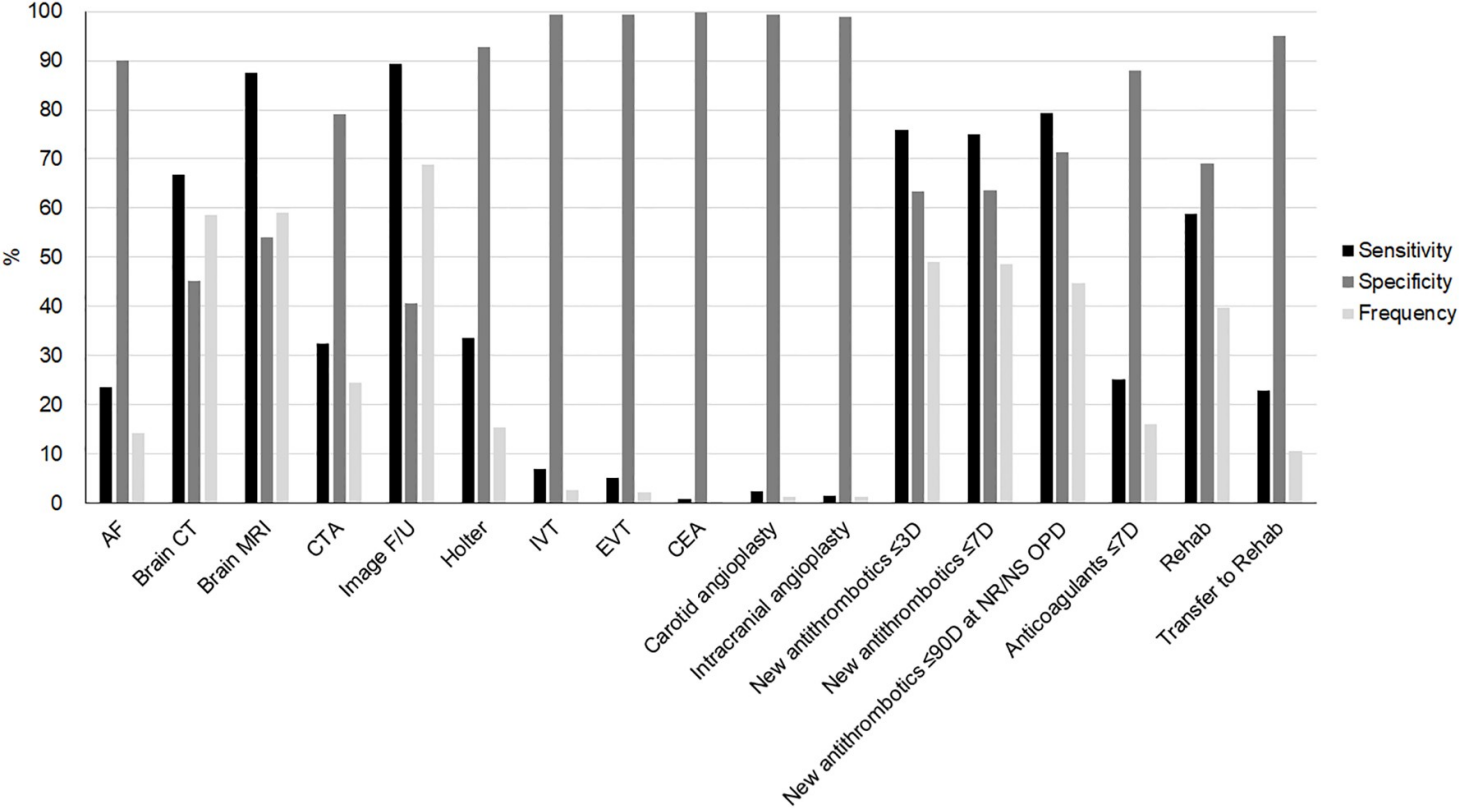
Sensitivity, specificity and frequency of key identifiers. Abbreviations: AF, atrial fibrillation; CTA, CT angiography; F/U, follow up; IVT, intravenous thrombolysis; EVT, endovascular treatment; CEA, carotid endarterectomy; 3D, 3 days; 7D, 7 days; 90D, 90 days; NR, Neurology; NS, Neurosurgery; OPD, out-patient department; Rehab, rehabilitation.

Among potential candidates for key identifiers, those with a sensitivity and specificity of less than 50% were excluded. In total, 17 key identifiers were selected (Fig 3). Key identifiers included one diagnosis code for AF and one claim code for evaluation of arrhythmia, four for brain imaging, two for reperfusion therapy, three for intervention or surgery, four for antithrombotic therapy, and two for rehabilitation. Frequencies of these key identifiers among the 40,443 episodes ranged from 0.4% to 80% (S1 Table). The sensitivity of the imaging-related key identifiers ranged from 32% to 91%, and the specificity ranged from 25% to 79%. MRI was more sensitive and specific than CT. Key identifiers related to reperfusion therapy and interventions showed a low sensitivity of less than 10% but a high specificity of more than 98%. *New antithrombotics* had a high sensitivity and specificity of approximately 70%. *Transfer to rehab* had a higher specificity but lower sensitivity than *rehab*.

**Fig 4:**
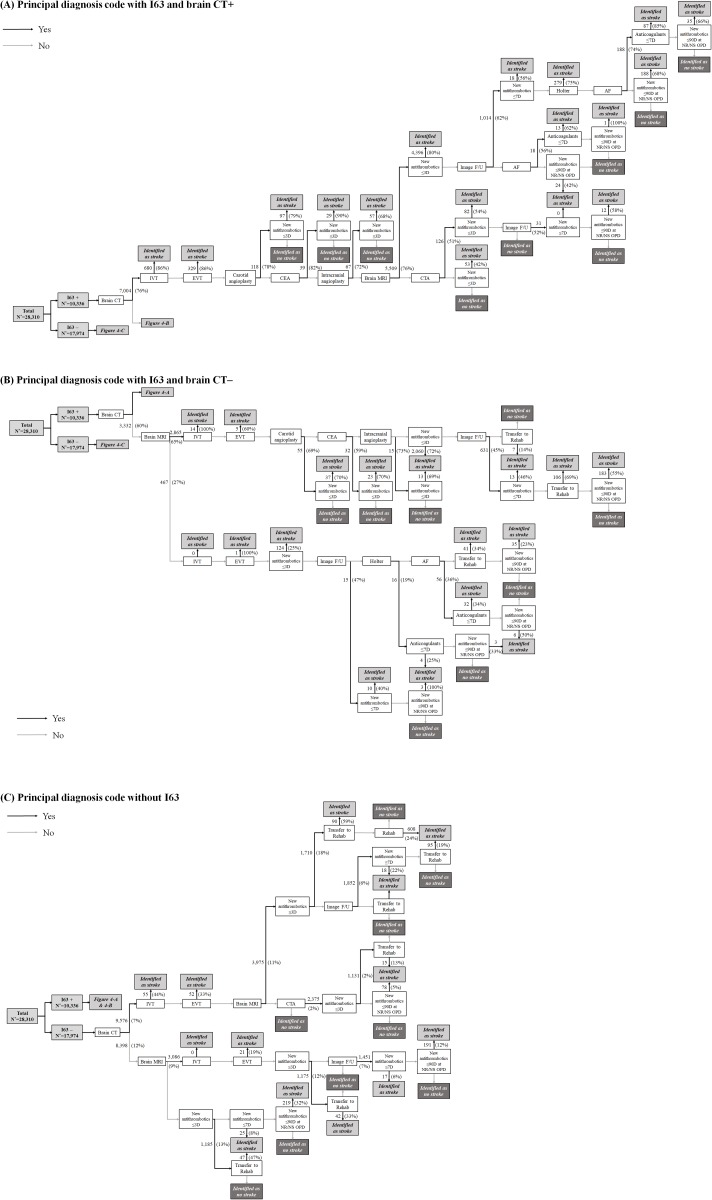
Stroke identification algorithm according to principal diagnosis code with (A) I63 and brain CT, (B) with I63 and brain CT, and (C) without I63. The number of cases selected by each step (the percent of the true AIS cases among the selected cases) is presented on each arrow.

The stroke identification algorithm was constructed with the combination of these 17 key identifiers according to the clinical flow of stroke care in practice (Fig 4). In total, 57 trajectories were generated. The algorithm began by distinguishing whether the principal diagnosis code was I63 (“I63+”, Fig 4A and 4B) or not (“I63–”, Fig 4C). After dividing the 40,443 episodes into the development (N = 28,310) and internal validation (N = 12,133) datasets with a ratio of 7:3, the algorithm was applied to each dataset.

Among I63+ cases (N = 10,336) from the development dataset, brain CT was performed in 7,004 cases (68%) (Fig 4A). Approximately 7% and 3% of I63+ cases were identified as AIS by IVT and EVT, respectively. Furthermore, using the combination of interventions-related identifiers (*carotid endarterectomy*, *carotid and intracranial angioplasty/stenting*) and *new antithrombotics*, 183 cases (2%) were identified as AIS. The combination of *new antithrombotics* and *brain MRI* or *brain CTA* followed, and 4,396 (43%) and 82 cases (0.8%) were selected, respectively. When the antithrombotic medication was not changed within 3 days despite *brain MRI*, we used the *image F/U* identifier with the suspicion of the lesion-negative TIA. Moreover, in the case of positive *image F/U*, *new antithrombotics within 7 days*, *Holter*, *AF*, *anticoagulants*, *and new antithrombotics within 90 days* were sequentially used to identify AIS. In the case of negative *image F/U*, *AF*, *anticoagulants*, *and new antithrombotics within 90 days* were also used. In the absence of antithrombotics despite positive *AF*, hemorrhagic transformation was considered the reason as to why antithrombotic medication could not be administered during hospitalization. Cases with *new antithrombotics within 90 days* at outpatient clinics were identified as stroke. The algorithm for I63+ cases without brain CT is presented in Fig 4B, in which the principles were similar to those of I63+ with brain CT, as described above.

In I63– cases (N = 17,974), 128 cases (0.7%) received reperfusion therapy (IVT or EVT) and were identified as AIS (Fig 4C). The algorithm was not different from that of I63+; however, interventions-related identifiers could not be applied due to clinical irrelevance. Moreover, the diagnostic accuracy of *new antithrombotics within 3 days* was not high and ranged from 12 to 18%. Therefore, the key identifier of *transfer to rehab* was added in the next step to improve accuracy with 47–59%.

The final sensitivity, specificity, and accuracy of the algorithm in the development dataset were 81.6%, 82.5%, and 82.2%, respectively (Table 2 and S2 Table). The negative predictive value of 90.6% was better than the positive predictive value of 68.3%. Internal validation with applying the same algorithm to the validation dataset (N = 12,133) showed similar results.

**Table 2:** Matched proportion in development and validation set.

	Sensitivity (%)	Specificity (%)	Accuracy (%)	PPV (%)	NPV (%)
Development set (N = 28,310)	81.6	82.5	82.2	68.3	90.6
Validation set (N = 12,133)	81.5	81.6	81.6	67.3	90.5

Abbreviations: PPV = positive predictive value; NPV = negative predictive value.

## Discussion

In this study, we developed a stroke identification algorithm based on the national claims data and updated and validated the algorithm through the linkage between the claims data and the multicenter stroke registry database, which has not been reported previously. Few previous studies using claims data validated their stroke identification process by chart review or direct interview, which inevitably led to the limitation of small sample sizes.[17, 19] Our study aimed to overcome this limitation through the linkage between claims data and a pre-validated, large-sized registry database.

This linkage between claims data and the large stroke registry can be a solution in a country like South Korea that has a universal coverage by one national health insurance service where the stroke registry is nationally representative. However, for its continuous usage, the algorithm also should be validated and updated continuously, as insurance claims are dependent on practice patterns and are heavily affected by reimbursement policies and financial incentives. It has been reported that in South Korea the stroke incidence based on stroke diagnosis codes from claims data increased sharply by 150% after expanding the coverage for reimbursement for brain MRIs in the same year.[26] As the diagnosis and treatment of stroke are evolving over time, the stroke identification algorithm using claims data should be adapted accordingly.

In a previous study evaluating the sensitivity and specificity of IVT in claims data for identifying stroke cases receiving IVT,[28] they were more than 90% in cases with the principal diagnosis code of I63. In the development dataset with principal or additional diagnosis code of I60–I64, we found 749 cases with IVT-related claims codes, and, of these, 83% were confirmed as AIS by the linkage with the registry database. No previous studies have reported on the validity of EVT-related claims codes. We found 387 cases with EVT codes, and 79% of these cases were confirmed as AIS. This indicates that reperfusion therapy-related claims codes can be used to identify AIS, especially when combined with stroke diagnosis codes or other stroke-related claims codes.

Among key identifiers related to antithrombotic medication, both the sensitivity and specificity were highest in *new antithrombotics within 90 days* at outpatient clinics, which means that, in the daily practice of South Korea, the change of antithrombotic medication occurs usually at outpatient clinics within 90 days from stroke onset. It is noteworthy that the probability of being true AIS is quite high in cases transferred from a neurology department to a rehabilitation department. This *transfer to rehab* identifier may be helpful in differentiating AIS cases from those hospitalized due to chronic stroke-related illness, although it should be confirmed in a future study.

In a few studies reporting the validity of operational criteria for identifying AIS, the diagnostic accuracy ranged from 43% to 64%.[15–17, 19] This may be attributed to those studies using only diagnosis codes of I60–I64 for identifying stroke cases. In this study, we defined 17 key identifiers, including ICD-10 diagnosis codes and claims codes related to diagnosis and treatment of AIS, and used their combinations to identify AIS. Our efforts improved the sensitivity and specificity of identifying AIS and successfully ruled out non-acute stroke cases with a negative predictive value of more than 90%.

The main problems in stroke research using claims data originate from the limitation of ICD-10 diagnosis codes. Diagnosis codes regarding stroke (I60-69) could not differentiate acute stroke patients from non-acute stroke survivors. A previous systematic review reported that, regarding the validity of diagnosis codes for any ischemic stroke, the positive predictive value was ≥ 82% and the sensitivity was ≥ 76% without considering the acuteness of stroke events.[29] However, in stroke research studies in which acute stroke is a primary concern, these diagnosis codes for any ischemic stroke cannot be used, because they include non-acute stroke, late effects of stroke, and ill-defined cerebrovascular disease.[29] Due to this problem, stroke diagnosis codes in the ICD-11 are recategorized as “cerebral ischemic stroke (8B11)” and “cerebrovascular disease with no acute cerebral symptom (8B21)”. Implementation of the ICD-11 is expected to begin after 2022.

Our study implicates that a stroke surveillance system using claims data with the linkage with a nationally representative stroke registry can be developed and is feasible in a country like South Korea that has a single-payer health insurance system with universal coverage. However, the following limitations should be noted. The major limitation is the lack of external validation. The algorithm should be tested outside of our stroke registry settings of university hospitals or regional stroke centers, which requires additional resources. Through this external validation process, our algorithm can be improved further. Representation may be another issue. There are 42 tertiary and 299 general hospitals in South Korea. We enrolled four tertiary and six general hospitals, which are approximately 10% and 2% of tertiary and general hospitals in Korea, respectively. Although stroke patients registered into the CRCS-K database were nationally representative regarding demographics,[30] other patient characteristics and diagnostic and therapeutic procedures of those recruited in the CRCS-K may be different from those outside of the CRCS-K. Again, in order to apply this algorithm to the entire population who were given stroke diagnosis codes, an update by external validation is mandatory. Lastly, a small portion of AIS cases that did not give consent or were admitted to departments other than neurology could be misclassified as non-AIS. However, the consent rate of 92.4% in this study was quite high, and our current Personal Information Protection Act does allow linkage using personal information without consent.

## Conclusions

In conclusion, through the linkage between national claims data and the nationwide multicenter stroke registry database, a stroke identification algorithm was developed and validated with acceptable sensitivity and specificity of above 80%. This algorithm may be useful to study stroke epidemiology at a national level in Korea, where all the residents are covered by the single-payer health insurance system, and their claims data were centered to one agency. However, further efforts to refine the algorithm are necessary.

## Supporting information

S1 Fig Clinical workflows of acute ischemic stroke patients in Korea. The key identifiers are remarked in bold, italic, and underlined form. Abbreviations: ER, emergency room; CTA, CT angiography; MRA, MR angiography; IVT, intravenous thrombolysis; EVT, endovascular treatment; GW, general wards; SU, stroke units; ICU, intensive care units; F/U, follow up; OPD, outpatient department.
(DOCX)

S1 Table Sensitivity, specificity and frequency of key identifiers. Abbreviations: AF, atrial fibrillation; CTA, CT angiography; F/U, follow up; IVT, intravenous thrombolysis; EVT, endovascular treatment; CEA, carotid endarterectomy; 3D, 3 days; 7D, 7 days; 90D, 90 days; NR, Neurology; NS, Neurosurgery; OPD, outpatient department; Rehab, rehabilitation.
(DOCX)

S2 Table Variables used for calculation of sensitivity, specificity and predictive value. Abbreviations: CRCS-K, Clinical Research Collaboration for Stroke in Korea; AIS, acute ischemic stroke; TP, true positive; FP, false positive; FN, false negative; TN, true negative.
(DOCX)
