# Supplementary material for: Development of stroke identification algorithm for claims data using the multicenter stroke registry database
Source: PLoS One. 2020 Feb 14;15(2):e0228997. doi: 10.1371/journal.pone.0228997 (PMC7021298; doi:10.1371/journal.pone.0228997)
Supplement: S1 Fig — The key identifiers are remarked in bold, italic, and underlined form. Abbreviations: ER, emergency room; CTA, CT angiography; MRA, MR angiography; IVT, intravenous thrombolysis; EVT, endovascular treatment; GW, general wards; SU, stroke units; ICU, intensive care units; F/U, follow up; OPD, outpatient department. (DOCX) [file pone.0228997.s001.docx]

**S1 Fig. Clinical workflows of acute ischemic stroke patients in Korea.**


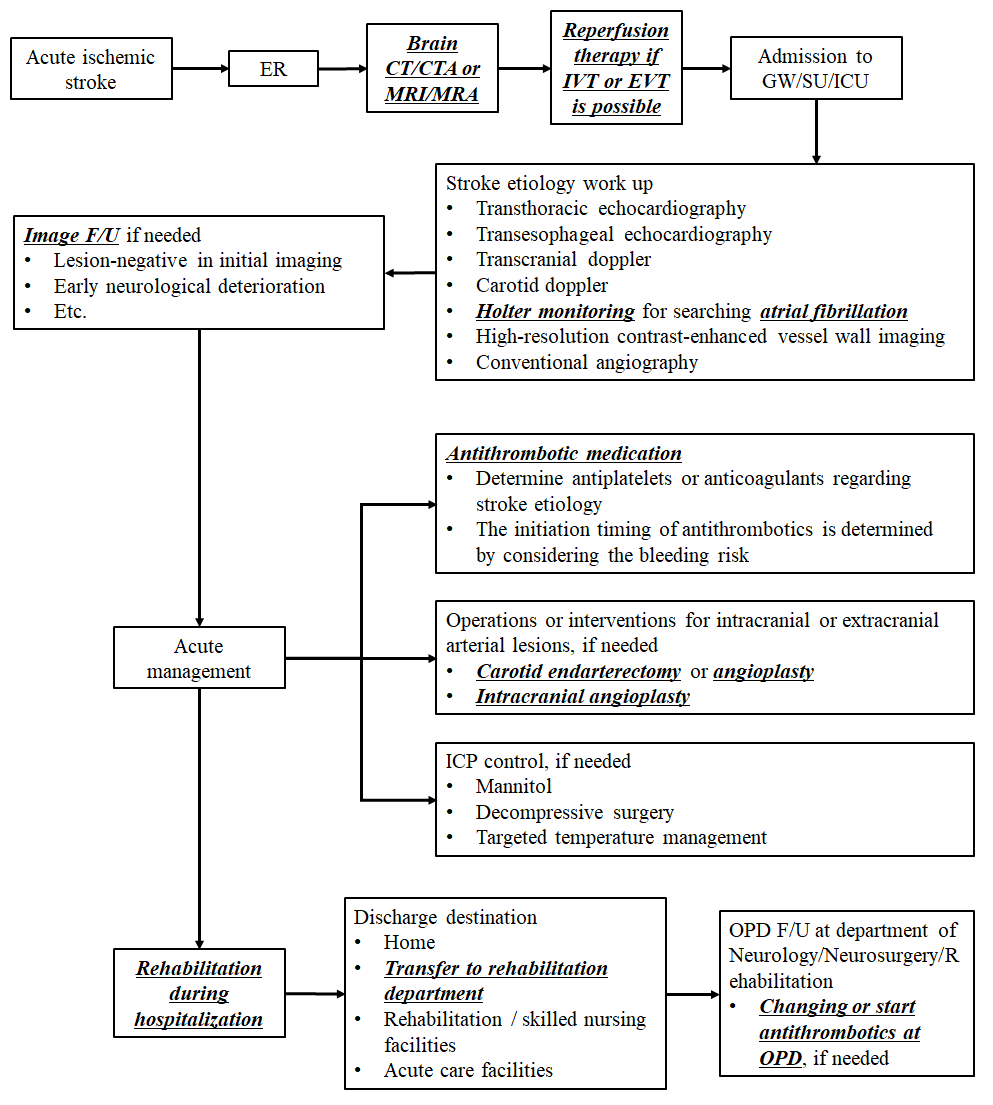


The key identifiers are remarked in bold, italic, and underlined form.

Abbreviations: ER, emergency room; CTA, CT angiography; MRA, MR angiography; IVT, intravenous thrombolysis; EVT, endovascular treatment; GW, general wards; SU, stroke units; ICU, intensive care units; F/U, follow up; OPD, outpatient department.
