# Supplementary material for: Development of stroke identification algorithm for claims data using the multicenter stroke registry database
Source: PLoS One. 2020 Feb 14;15(2):e0228997. doi: 10.1371/journal.pone.0228997 (PMC7021298; doi:10.1371/journal.pone.0228997)
Supplement: S1 Table — Abbreviations: AF, atrial fibrillation; CTA, CT angiography; F/U, follow up; IVT, intravenous thrombolysis; EVT, endovascular treatment; CEA, carotid endarterectomy; 3D, 3 days; 7D, 7 days; 90D, 90 days; NR, Neurology; NS, Neurosurgery; OPD, outpatient department; Rehab, rehabilitation. (DOCX) [file pone.0228997.s002.docx]

**S1 Table. Sensitivity, specificity and frequency of key identifiers.**

|  | Sensitivity (%) | Specificity (%) | Accuracy (%) | Frequency (%) |
| --- | --- | --- | --- | --- |
| AF | 23.6 | 90.0 | 69.0 | 14.3 |
| Brain CT | 66.9 | 45.3 | 52.1 | 58.6 |
| Brain MRI | 87.4 | 54.1 | 64.6 | 59.1 |
| CTA | 32.4 | 79.1 | 64.3 | 24.5 |
| Image F/U | 89.3 | 40.7 | 56.1 | 68.8 |
| Holter | 33.6 | 92.8 | 74.1 | 15.5 |
| IVT | 7.0 | 99.4 | 70.1 | 2.7 |
| EVT | 5.2 | 99.3 | 69.5 | 2.1 |
| CEA | 0.8 | 99.8 | 68.5 | 0.4 |
| Carotid angioplasty | 2.4 | 99.3 | 68.7 | 1.2 |
| Intracranial angioplasty | 1.6 | 98.8 | 68.1 | 1.3 |
| New antithrombotics ≤3D | 75.8 | 63.4 | 67.3 | 49.0 |
| New antithrombotics ≤7D | 75.1 | 63.6 | 67.2 | 48.6 |
| New antithrombotics ≤90D at NR/NS OPD | 79.4 | 71.3 | 73.8 | 44.8 |
| Anticoagulants ≤7D | 25.1 | 88.0 | 68.1 | 16.1 |
| Rehab | 58.8 | 69.1 | 65.8 | 39.7 |
| Transfer to Rehab | 23.0 | 95.1 | 72.3 | 10.6 |

Abbreviations: AF, atrial fibrillation; CTA, CT angiography; F/U, follow up; IVT, intravenous thrombolysis; EVT, endovascular treatment; CEA, carotid endarterectomy; 3D, 3 days; 7D, 7 days; 90D, 90 days; NR, Neurology; NS, Neurosurgery; OPD, outpatient department; Rehab, rehabilitation.
