# Supplementary material for: Development of stroke identification algorithm for claims data using the multicenter stroke registry database
Source: PLoS One. 2020 Feb 14;15(2):e0228997. doi: 10.1371/journal.pone.0228997 (PMC7021298; doi:10.1371/journal.pone.0228997)
Supplement: S2 Table — Abbreviations: CRCS-K, Clinical Research Collaboration for Stroke in Korea; AIS, acute ischemic stroke; TP, true positive; FP, false positive; FN, false negative; TN, true negative. (DOCX) [file pone.0228997.s003.docx]

**S2 Table. Variables used for calculation of sensitivity, specificity and predictive value.**

|  | Gold standard (CRCS-K registry database) | | Total |
| --- | --- | --- | --- |
|  | True AIS | No stroke |  |
| Algorithm (claims database) |  |  |  |
| Identified as stroke | TP | FP | TP + FP |
| Identified as no stroke | FN | TN | FN + TN |
| Total | TP + FN | FP + TN | TP + FP + FN + TN |

Abbreviations: CRCS-K, Clinical Research Collaboration for Stroke in Korea; AIS, acute ischemic stroke; TP, true positive; FP, false positive; FN, false negative; TN, true negative.
